# Alleviation of behavioral hypersensitivity in mouse models of inflammatory pain with two structurally different casein kinase 1 (CK1) inhibitors

**DOI:** 10.1186/1744-8069-10-17

**Published:** 2014-03-10

**Authors:** Takashi Kurihara, Eri Sakurai, Masayasu Toyomoto, Isao Kii, Daisuke Kawamoto, Toshihide Asada, Tsutomu Tanabe, Megumu Yoshimura, Masatoshi Hagiwara, Atsuro Miyata

**Affiliations:** 1 Department of Pharmacology, Graduate School of Medical and Dental Sciences, Kagoshima University, 8-35-1 Sakuragaoka, Kagoshima City, Kagoshima 890-8544, Japan; 2 Department of Pharmacology and Neurobiology, Graduate School of Medicine, Tokyo Medical and Dental University, 1-5-45 Yushima, Bunkyo-ku, Tokyo 113-8519, Japan; 3 Department of Anatomy and Developmental Biology, Graduate School of Medicine, Kyoto University, Yoshida-Konoe-cho, Sakyo-ku, Kyoto 606-8501, Japan; 4 Graduate School of Health Sciences, Kumamoto Health Science University, 325 Izumi-machi, Kumamoto 861-5598, Japan

**Keywords:** Allodynia, Carrageenan, Complete Freund’s adjuvant, CFA, Hyperalgesia, Whole-cell patch-clamp

## Abstract

**Background:**

The phylogenetically highly conserved CK1 protein kinases consisting of at least seven isoforms form a distinct family within the eukaryotic protein kinases. CK1 family members play crucial roles in a wide range of signaling activities. However, the functional role of CK1 in somatosensory pain signaling has not yet been fully understood. The aim of this study was to clarify the role of CK1 in the regulation of inflammatory pain in mouse carrageenan and complete Freund’s adjuvant (CFA) models.

**Results:**

We have used two structurally different CK1 inhibitors, TG003 and IC261. TG003, which was originally identified as a cdc2-like kinase inhibitor, had potent inhibitory effects on CK1 isoforms *in vitro* and in cultured cells. Intrathecal injection of either TG003 (1-100 pmol) or IC261 (0.1-1 nmol) dose-dependently decreased mechanical allodynia and thermal hyperalgesia induced by carrageenan or CFA. Bath-application of either TG003 (1 μM) or IC261 (1 μM) had only marginal effects on spontaneous excitatory postsynaptic currents (sEPSCs) recorded in the substantia gelatinosa neurons of control mice. However, both compounds decreased the frequency of sEPSCs in both inflammatory pain models.

**Conclusions:**

These results suggest that CK1 plays an important pathophysiological role in spinal inflammatory pain transmission, and that inhibition of the CK1 activity may provide a novel strategy for the treatment of inflammatory pain.

## Background

Increased sensitivity to both noxious and non-noxious stimuli is a hallmark of persistent pain states following tissue injury and inflammation. This hypersensitivity is associated with both peripheral and spinal neuronal plasticities, leading to a reduction of activation threshold in peripheral nociceptive sensory neurons in the dorsal root ganglion (DRG) and trigeminal ganglion, as well as an increase in the synaptic activity between sensory nerve endings and second-order neurons in the spinal dorsal horn [1-3]. Inflammatory pain is typically treated with opioids and non-steroidal anti-inflammatory drugs such as cyclooxygenase 2 inhibitors. However, these treatments are currently limited by well-known side effects. Acute opioid treatment produces respiratory depression, sedation, nausea, constipation and vomiting, and long-term treatment with opioids and cyclooxygenase 2 inhibitors is associated with the development of addiction and cardiovascular defects, respectively. Thus, chronic pain associated with inflammation is still difficult to treat, and development of new strategies leading to pharmacological treatment of inflammatory pain is eagerly awaited.

Casein kinases (CK) were one of the first serine/threonine protein kinases to be identified and characterized in the 1970s [4-7]. Two distinct CK activities were recognized, leading to the identification of two different kinases, CK1 and CK2. Whereas CK2 belongs to the CMGC (cyclin-dependent kinase, mitogen-activated protein kinase, glycogen synthase kinase, CDC-like kinase) group, CK1 forms one of the eight major groups of protein kinases identified in the human and mouse genomes [8,9]. The CK1 family consists of several isoforms that include CK1α, CK1γ1-CK1γ3, CK1δ, and CK1ϵ and their various splice variants. CK1 is present in different cell types and in subcellular compartments, including the plasma membrane, cytosol, and nucleus. The widespread distribution of CK1 suggests important regulatory roles of this protein kinase. At present, CK1 has been implicated in diverse biological processes including circadian rhythms, membrane trafficking, cytoskeleton maintenance, DNA and RNA metabolism [4-6]. However, the function of CK1 in the somatosensory pathway has not yet been fully examined.

IC261 is a commonly used and commercially available CK1 inhibitor, which is reported to be relatively specific for CK1δ and ϵ isoforms [10], although some of its effects are likely to be independent from CK1 inhibition [11]. Previously we demonstrated that intrathecal administration of IC261 effectively reversed neuropathic pain-like behavior in mice [12]. TG003 originally identified as a cdc2-like kinase (Clk) inhibitor [13,14], has recently been shown to inhibit CK1δ and ϵ activities equally to, or more potently than IC261 *in vitro*[15,16]. In this study, we examined the effects of these two structurally different CK1 specific inhibitors on inflammatory pain induced by peripheral treatment of carrageenan or CFA. A preliminary report of this study has been presented elsewhere [17].

## Results

### Evaluation of TG003 as an inhibitor of CK1 family members

**Figure 1:**
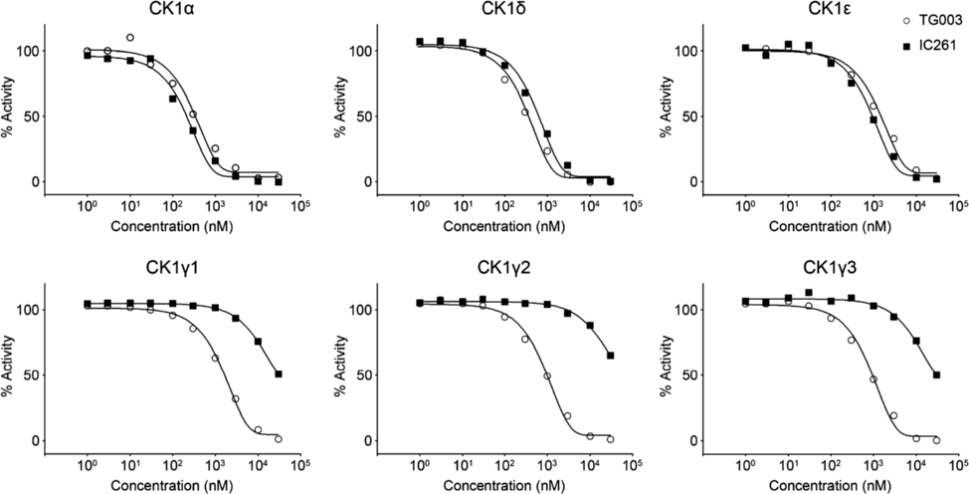
**TG003 suppresses kinase activity of CK1 family members *in vitro*.** Recombinant CK1 family members were incubated with the substrate peptide CKtide in the presence of different concentrations of TG003 and IC261. TG003 (open circle) and IC261 (closed square) inhibited CK1 family members in a dose-dependent manner. The IC_50_ values of TG003 on the kinase activity of CK1α, δ, ϵ, γ1, γ2 and γ3were 0.33, 0.34, 1.4, 1.5, 0.93 and 0.88 μM, respectively. The IC_50_ values of IC261 were 0.19, 0.60, 0.86, > 30, > 30 and > 30 μM, respectively. Representative dose-response curves with Hill slope are shown. The results are presented as an average of duplicated experiments.

We first verified the inhibitory effect of TG003 on the enzymatic activity of CK 1 family members and compared it with that of IC261. Recombinant CK1α, δ, ϵ, γ1, γ2 or γ3 were incubated with the substrate peptide CKtide in the presence of different concentrations of TG003 or IC261, respectively. Both small molecules inhibited CK1 family members in a dose-dependent manner (Figure 1). Inhibition of CK1α, δ and ϵ by TG003 were equivalent to that by IC261. On the other hand, the inhibitory activities of TG003 on the kinase activity of CK1γ1, γ2 and γ3 were 20-fold or more higher than those of IC261 (Figure 1). These results indicate that TG003 and IC261 are able to suppress the kinase activity of broad CK1 family members equally.

### TG003 inhibited CK1δ and ϵ-induced nuclear translocation of PER3

**Figure 2:**
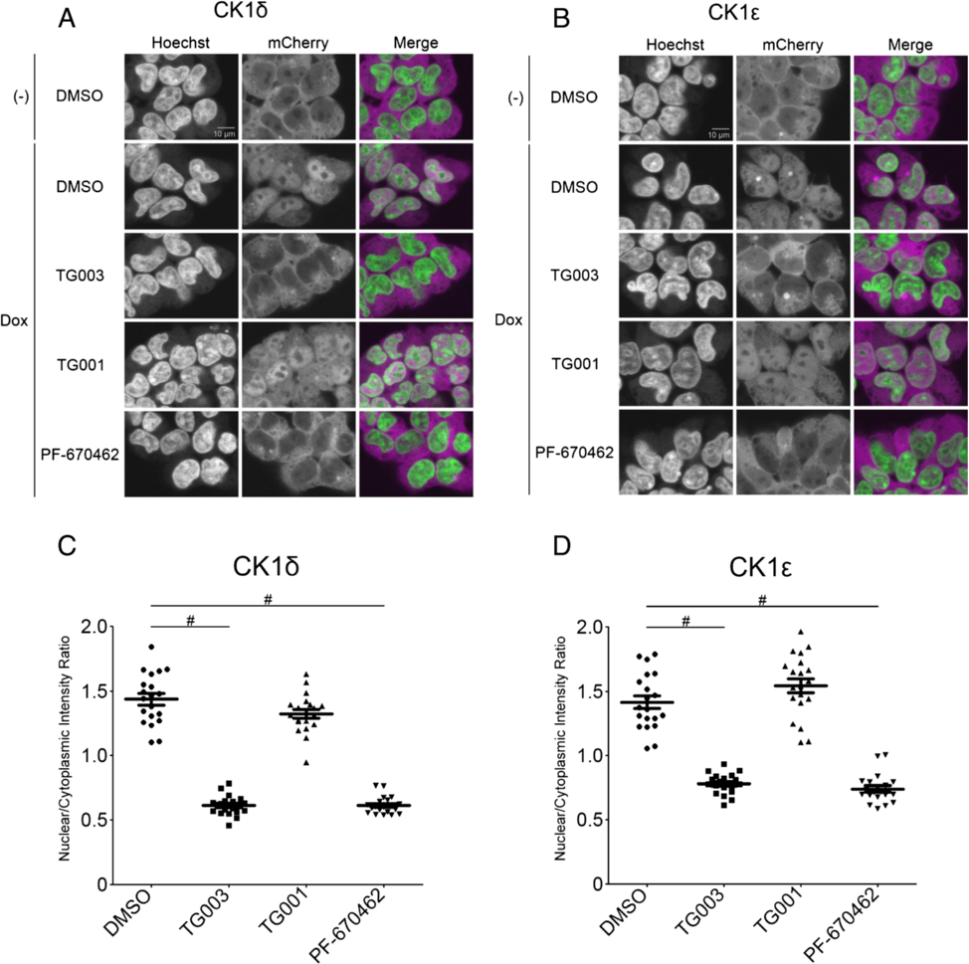
**TG003 inhibits CK1δ/ϵ-induced nuclear translocation of PER3. (A, B)** Localization of mCherry-PER3 in HEK293 cells expressing CK1δ **(A)** or CK1ϵ **(B)**. Prior to doxycycline (Dox)-induced expression of CK1δ/ϵ for 8 hours, the cells were treated with vehicle control, TG003, TG001, or PF-670462 for 1 hour. The treated cells were fixed and stained with Hoechst33342 to define nucleus. Representative images are shown. **(C, D)**. Quantification of the nuclear/cytoplasmic fluorescence intensity ratio. The data are mean ± SEM (n = 20). ^#^*P* < 0.0001 (Student’s *t*-test).

We next examined whether TG003 inhibits CK1δ and ϵ in living cells. To quantify the kinase activity of CK1δ and ϵ, we utilized CK1-induced nuclear translocation of PER3. PER3 is one of the mammalian homologue of *period*, which is a core molecular component of circadian rhythm and is involved in transcription-translation oscillatory feedback loops on the molecular level in the hypothalamic suprachiasmatic nucleus, the master pacemaker regulating circadian rhythms [4,18]. Phosphorylation of PER3 by CK1δ and/or CK1ϵ in the cytoplasm induces their translocation to the nucleus [19]. We constructed a constitutive expression vector of PER3 fused with mCherry (mCherry-PER3), and cloned both CK1δ and CK1ϵ under control of a doxycycline-inducible promoter. HEK293 cells were transfected with these recombinant vectors, and stable cell lines expressing both mCherry-PER3 and CK1 were established (Figure 2A, B). In the absence of doxycycline, fluorescence signals of mCherry-PER3 were mainly detected in the cytoplasm. Treatment with doxycycline for 8 hours, which triggered the expression of CK1δ and ϵ, respectively, induced nuclear accumulation of mCherry-PER3. Co-administration of TG003 with doxycycline inhibited the nuclear translocation of mCherry-PER3. Similarly, the CK1 specific small molecule inhibitor, PF-670462, which was used as positive control [20], also inhibited the nuclear translocation (Figure 2A, B). On the other hand, a structurally similar compound TG001 [13], which possesses no inhibitory effect on CK1δ and ϵ in the *in vitro* assay (data not shown), did not prevent the nuclear translocation of mCherry-PER3. To quantify the inhibitory effects on the nuclear translocation, we measured the fluorescence intensities of mCherry in the nucleus and cytoplasm by compartmental analysis using Cellomics BioApplications software for 20 images of each one, and calculated the mCherry-PER3 nuclear/cytoplasmic ratio as described in Methods. The ratio was significantly decreased upon TG003 or PF-670462, compared to that upon TG001 or vehicle treatment (Figure 2C, D), indicating that TG003 inhibits the function of CK1δ and ϵ in living cells.

### Intrathecal injection of IC261 or TG003 attenuated acute and persistent inflammatory pain behaviors

**Figure 3:**
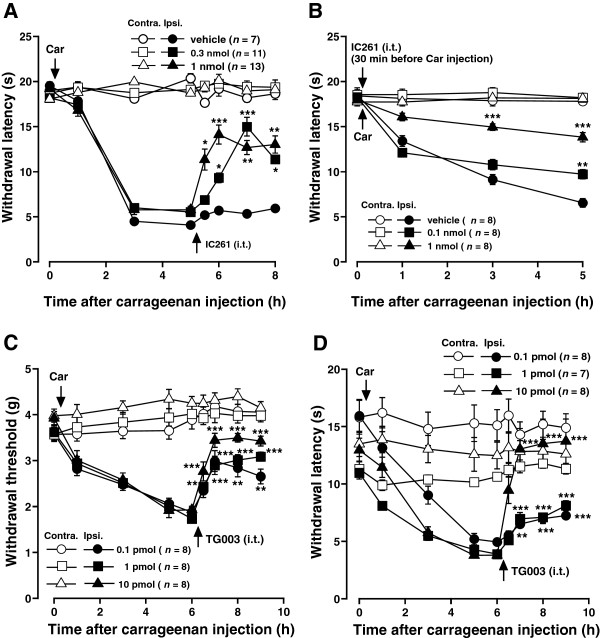
**Effects of CK1 inhibitors on carrageenan-induced acute inflammatory pain behaviors. (A, B)** Effects of intrathecal injection of IC261 on carrageenan (Car)-induced thermal hyperalgesia. IC261 was injected 5 hour after **(A)** or 30 min before **(B)** carrageenan injection. **(C, D)** Effects of intrathecal injection of TG003 on carrageenan (Car)-induced mechanical allodynia **(C)** and thermal hyperalgesia **(D)**. Paw withdrawal threshold to mechanical stimulation and paw withdrawal latency to thermal stimuli are plotted against the time after carrageenan injection into a hindpaw. Data are mean ± SEM. ^*^P < 0.05, ^**^P < 0.01, ^***^P < 0.001, compared with pre-drug data (one-way ANOVA followed by Dunnett’s post hoc test).

**Figure 4:**
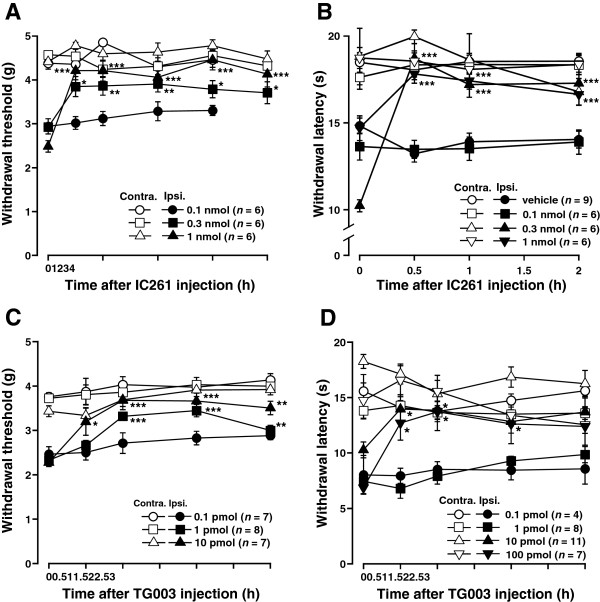
**Effects of CK1 inhibitors on CFA-induced persistent inflammatory pain behaviors. (A, B)** Effects of intrathecal injection of IC261 on CFA-induced mechanical allodynia **(A)** and thermal hyperalgesia **(B)**. **(C, D)** Effects of intrathecal injection of TG003 on CFA-induced mechanical allodynia **(C)** and thermal hyperalgesia **(D)**. Paw withdrawal threshold to mechanical stimulation and paw withdrawal latency to thermal stimuli are plotted against the time after intrathecal injection into IC261 or TG003. CFA was injected 3 days before. Data are mean ± SEM. ^*^P < 0.05, ^**^P < 0.01, ^***^P < 0.001, compared with pre-drug (at 0 hour) data (one-way ANOVA followed by Dunnett’s post hoc test).

To investigate whether CK1 is involved in the inflammatory pain states, we evaluated the effects of IC261 or TG003 in mouse models of inflammatory pain. I.t. injections of IC261 (0.1-1 nmol) or TG003 (0.1-100 pmol) dose-dependently increased both withdrawal threshold and withdrawal latency of the hind paw ipsilateral to carrageenan or CFA-induced inflammation (Figures 3 and 4). Spinal preemptive treatment of IC261 also dose-dependently attenuated the development of thermal hyperalgesia induced by carrageenan (Figure 3B). Thus, blocking the CK1 activity at the spinal level appeared to be effective in reduction of inflammation-induced mechanical allodynia and thermal hyperalgesia. The maximum effects were observed 0.5-1 hour after the injections of both inhibitors and significant analgesic effects were still observed 3-4 hours after the injection of the highest doses used in this study (Figures 3 and 4). These inhibitors had no significant effects on the contralateral hind paw (Figures 3 and 4). I.t. injection of vehicle (1% DMSO in saline) used as a solvent for the drugs did not show any effects (Figures 3A and 4B, data not shown).

### Carrageenan- and CFA-induced inflammation did not upregulate CK1α, δ and ϵ protein expression

We next examined the protein expression levels of CK1α, δ and ϵ protein in the spinal cord (L4-6) and DRGs (L4-6) by immunoblot analyses. Expression of the three CK1 isoforms were not significantly altered in both spinal cord (carrageenan model: CK1α, 95.6 ± 11.2% of control, n = 6; CK1δ, 143.4 ± 25.0%, n = 11; CK1ϵ, 101.7 ± 9.25%, n = 6; CFA model : CK1α, 108.7 ± 18.3%, n = 6; CK1δ, 99.7 ± 13.7%, n = 11; CK1ϵ, 93.6 ± 10.8%, n = 6) and DRGs (carrageenan model: CK1α, 111.4 ± 23.2% of control, n = 6; CK1δ, 125.4 ± 33.4%, n = 7; CK1ϵ, 109.8 ± 23.1%, n = 6; CFA model: CK1α, 92.4 ± 18.3% of control, n = 6; CK1δ, 102.2 ± 4.99%, n = 7; CK1ϵ, 96.8 ± 11.0%, n = 6) after carrageenan (6 hours)- or CFA (3 days)-treatment, respectively (see also Additional file 1).

### IC261 and TG003 decreased the frequency of sEPSCs in inflammatory pain model mice

To explore the mechanism of the antinociception induced by IC261 or TG003 at the spinal level, we prepared L5 spinal cord slice preparation from adult mice (7-10 weeks old) and performed patch-clamp recordings in lamina II SG neurons ipsilateral to carrageenan, CFA, or vehicle injection [21-23]. The SG neurons of the spinal dorsal horn play an important role in the transmission and modulation of nociceptive information from the periphery to the CNS [24-26], and is one of the key sites generating synaptic plasticity (central sensitization) after tissue injury [3,26,27]. Such plasticity is exhibited in part as changes in spontaneous excitatory and inhibitory postsynaptic currents (sEPSCs and sIPSCs, respectively), which could point out both presynaptic mechanisms (frequency changes) and postsynaptic mechanisms (amplitude changes) [28-32].

We first examined the passive membrane properties of SG neurons. All SG neurons examined had resting potentials more negative than -50 mV in control and two inflammatory pain model mice. No differences were found in the resting membrane potential and input membrane resistance among the groups (Additional file 2).

Next we characterized sEPSCs, recorded under voltage-clamp at a holding potential of -70 mV, from control and inflamed mice (Additional file 3A). The mean amplitude of sEPSCs was not significantly different among the groups. The mean frequency of sEPSCs, on the other hand, was significantly different. We found that the average frequency but not the amplitude of sEPSCs was significantly increased in mice inflamed with CFA 3 days before, although carrageenan inflammation did not increase both the average sEPSC frequency and amplitude 6 hours after the injection.

We further examined sIPSCs from control and inflamed mice SG neurons at holding potentials of 0 mV (Additional file 3B). Although the mean amplitude of sIPSCs was not different among the groups, the mean frequency of sIPSCs was significantly reduced in CFA groups.

**Figure 5:**
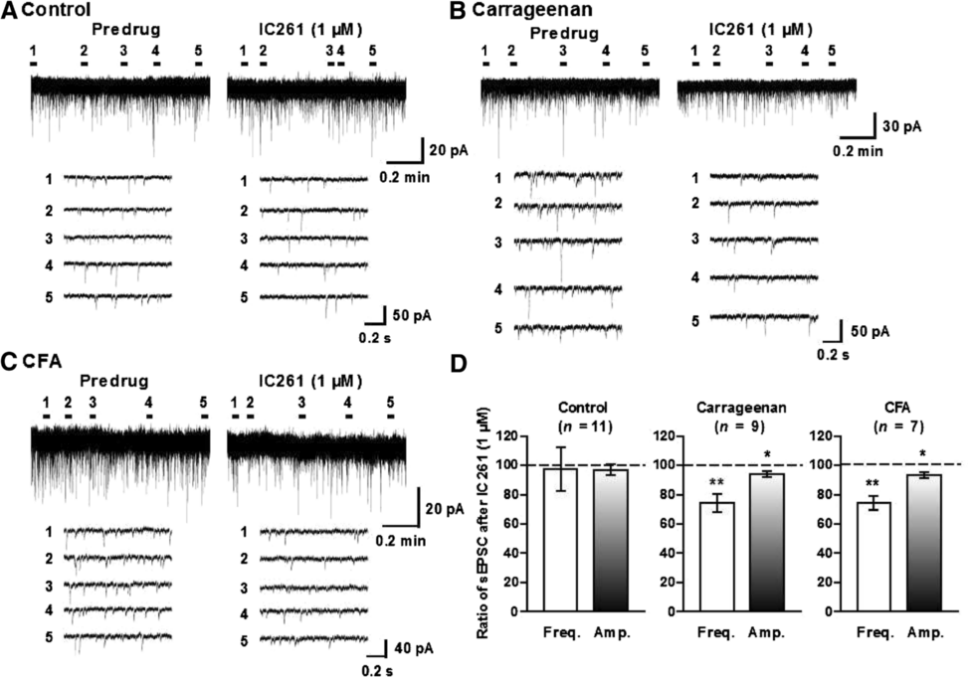
**IC261 decreased the mean frequency and amplitude of sEPSCs in inflammatory pain model mice.** Representative traces of sEPSCs in SG neurons of the spinal cord slices from naïve control **(A)**, carrageenan **(B)**- and CFA **(C)**-inflamed mice showing the effects of IC261 (1 μM). Lower five traces represent sEPSCs at five given points in time presented above the upper trace, and are shown in an expanded time scale. **(D)** Summary of results, testing the effects of IC261 on the sEPSC frequencies and amplitudes The percentage compared to pre-drug response (as 100%) was shown as% control. ^*^P < 0.05, ^**^P < 0.01, compared with pretreatment control (Student’s *t* test).

**Figure 6:**
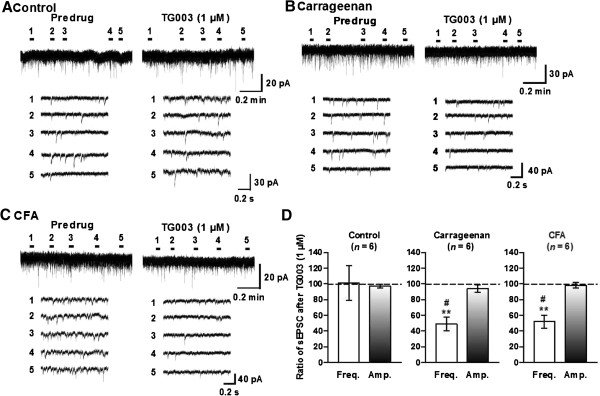
**TG003 decreased the mean frequency, but not the amplitude of sEPSCs in inflammatory pain model mice.** Representative traces of sEPSCs in SG neurons of the spinal cord slices from naïve control **(A)**, carrageenan **(B)**- and CFA **(C)**-inflamed mice showing the effects of TG003 (1 μM). Lower five traces represent sEPSCs at five given points in time presented above the upper trace, and are shown in an expanded time scale. **(D)** Summary of results, testing the effects of TG003 on the sEPSC frequencies and amplitudes. The percentage compared to pre-drug response (as 100%) was shown as % control. ^**^P < 0.01, compared with pretreatment control; ^#^*P* < 0.05, compared with the effect of IC261 (see Figure 5) (Student’s *t* test).

**Figure 7:**
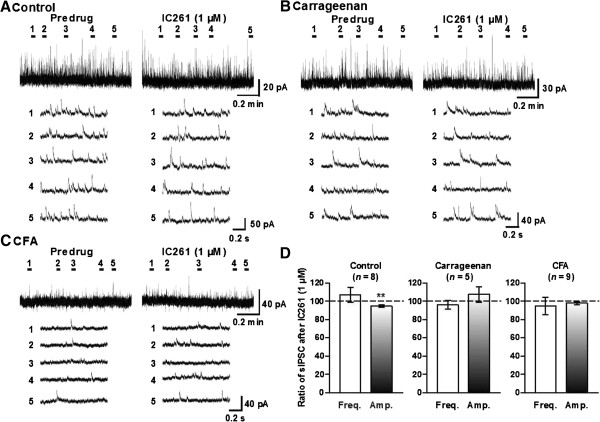
**IC261 had no effects on the sIPSCs in inflammatory pain model mice.** Representative traces of sIPSCs in SG neurons of the spinal cord slices from naïve control **(A)**, carrageenan **(B)**- and CFA **(C)**-inflamed mice showing the effects of 1 μM IC261. Lower five traces represent sIPSCs at five given points in time presented above the upper trace, and are shown in an expanded time scale. **(D)** Summary of results, testing the effects of IC261 on the mean frequencies and amplitudes of sIPSCs. The percentage compared to pre-drug response (as 100%) was shown as% control. ^**^P < 0.01, compared with pretreatment control (Student’s *t* test).

We then examined the possibility that the observed effects of the two CK1 inhibitors originate from the regulation of the excitatory and inhibitory synaptic transmission in lamina II of inflamed mice. Superfusion of spinal cord slices from control mice either treated with IC261 (1 μM) or TG003 (1 μM) altered neither the frequency nor the amplitude of sEPSCs (Figures 5A and 6A). However, both CK1 inhibitors significantly suppressed sEPSCs recorded from carrageenan (Figures 5B and 6B)- or CFA (Figures 5C and 6C)-treated mice. It should be noted, however, that the inhibitory effects on sEPSC frequencies were much more dramatic than those on sEPSC amplitudes (Figures 5D and 6D), and furthermore, the magnitude of inhibitory effects induced by TG003 on sEPSC frequencies was significantly higher than that induced by IC261 (Figures 5D and 6D) in both carrageenan and CFA treated animals. On the other hand, IC261 (1 μM) did not affect sIPSCs from the inflamed mice, although slight but significant reduction of the mean amplitude of control sIPSCs was observed (Figure 7).

## Discussion

The present study showed for the first time that the two structurally different CK1 inhibitors effectively reversed mechanical allodynia and thermal hyperalgesia induced by acute or persistent hindpaw inflammation. From *in vitro* whole-cell patch-clamp studies, a part of the analgesic mechanisms was suggested to be due to the inhibitory effects of the CK1 inhibitors on excitatory synaptic transmission within SG neurons of the inflamed mice.

### Pharmacological properties of IC261 and TG003

In this study we clarified that both IC261 and TG003 equally blocked CK1α, δ and ϵ activities. We also identified that TG003 effectively blocked activities of CK1γ isoforms. IC261 was initially reported as a selective CK1δ/ϵ inhibitor which blocked CK1δ and ϵ enzymatic activities more potently than CK1α activity [10]. However, our *in vitro* kinase assay and a recent report [16] indicated that IC261 exerted comparable inhibitory effects against CK1α, δ and ϵ isoforms, but inhibitory effects on three CK1γ isoforms were relatively weak. In contrast, TG003 demonstrated almost equal inhibitory effects among CK1 isoforms. Results from our preliminary screening experiments and reports from other groups [15,16] suggested that common targets for IC261 and TG003 are CK1α, δ and ϵ at this moment. Although relative importance of each CK1 isoform in the allodynia and hyperalgesia remains to be determined, CK1 might play an important role for the development and maintenance of inflammatory pain.

One important finding of this study is that TG003 produced antinociceptive effects on both carrageenan- and CFA-induced inflammatory pain models at lower doses than IC261. This difference may be due to the fact that TG003 also blocks CK1γ isoforms and Clks. In particular, IC_50_ values of TG003 against Clk1 and 4 isoforms (15-20 nM) [13] are smaller than those against CK1 isoforms. However, it remains to be determined whether activation of CK1γ isoforms and/or Clks significantly contributes to the pathogenic mechanism of pain. In addition, we could not exclude the possibility that other CK1-independent effects of TG003 and/or IC261 might affect the antinociceptive effects.

It would be noteworthy that TG003 preferentially alleviated mechanical allodynia than thermal hyperalgesia in both carrageenan and CFA models. Although 1 pmol of TG003 did not affect CFA-induced thermal hyperalgesia, the same dosage of TG003 significantly reversed CFA-induced mechanical allodynia. IC261, on the other hand, was shown to be equally effective on both mechanical allodynia and thermal hyperalgesia in the present inflammatory pain models, as well as in our previously described spinal nerve injury model [12]. The reason of this difference is currently unknown and further rigorous studies would be necessary to evaluate the pharmacological profiles of TG003 and IC261.

### Inhibition of pain-related synaptic plasticity by the CK1 inhibitors

Since intrathecal injection of these CK1 inhibitors reversed both mechanical and thermal nociceptive behaviors after peripheral inflammation, we investigated whether bath application of these CK1 inhibitors affects on sEPSCs and/or sIPSCs by using the whole-cell patch-clamp method in SG neurons of adult spinal cord slices.

First, we characterized the effects of carrageenan- or CFA-induced peripheral inflammation on the sEPSCs and sIPSCs. In general accordance with previous reports [28,33,34], we found that CFA inflammation for 3 days elicited significant increase in mean frequency of sEPSCs, and significant decrease of mean frequency, but not amplitude, of sIPSCs. In contrast, significant changes in frequencies and amplitudes of sEPSCs and sIPSCs were not observed 6 hours after carrageenan injection, which may be consistent with the previous report showing no alteration in frequencies and amplitudes of miniature EPSCs and IPSCs 1-2 days after carrageenan inflammation in immature rats [35]. One apparent difference between our present data and the previous report using mice CFA model [28] is that we could not detect significant increase in the mean amplitude of sEPSCs after CFA inflammation. The reason for this difference is at present unknown, but this might be due to the difference (this study vs. [28]) of strain (C57BL6/J vs. CD1), age (7-10 weeks old vs. 4-6 weeks old) or duration after CFA injection (3 days vs. 1 day).

More importantly, we found that bath-application of IC261 or TG003 had no effects on sEPSCs from control animals, but carrageenan and CFA inflammation turned the CK1 inhibitors effective in decreasing the mean frequencies of their respective sEPSCs. Since we did not characterize the SG neurons we recorded by anatomical and more detailed electrophysiological criteria [36,37] in this study, it is currently difficult to discuss possible involvement of CK1 in the superficial dorsal horn synaptic circuits. However, it may be worth noting here that both IC261 and TG003 exerted relatively consistent inhibitory effects on sEPSCs in the inflammatory pain models. In any case, this observation suggests that the nature of sEPSCs recorded in inflamed mice seems to be very different from those found in control animals. Our previous report also demonstrated similar specific inhibitory effects of IC261 on excitatory responses in dorsal horn elicited by dorsal root electrical stimulation only in spinal nerve injured but not in sham operated mice [12]. These results also seem to be consistent with the facts that these CK1 inhibitors dampen inflammatory (this study) and neuropathic (our previous study) pain-like behaviors without showing any appreciable effects on contralateral hindpaws.

Interestingly, we noticed a significant difference between IC261 and TG003 on inflamed mice, that is, 1 μM of TG003 had more potently inhibited the mean frequency of sEPSCs than the same concentration of IC261. At present, we could only speculate that the difference in their potencies might be derived from the distinct actions of TG003 on CK1 isoforms and/or Clks as described above, but further study is needed to verify this possibility.

The preferential inhibitory effects of IC261 and TG003 on the frequency of sEPSCs might suggest pre- rather than post-synaptic site of action of these molecules in SG synapses and this inhibitory modulation would contribute the antinociceptive effects on inflamed mice. It is generally believed that changes in the frequency and amplitude of sEPSCs are mediated by respective pre- and post-synaptic mechanisms [28-32]. We previously suggested that similar mechanism would be involved in the antinociceptive effects of CK1 inhibitors on neuropathic pain-like behaviors [12]. CK1 isoforms were shown to be associated with cytosolic vesicles including small synaptic vesicles and to phosphorylate several small synaptic vesicle-associated proteins in neuronal cells [38-40], suggesting a possible involvement of CK1 in the synaptic vesicle exocytosis [5,40].

At least CK1δ [41] and ϵ [12,42] proteins are shown to be expressed in mouse spinal dorsal horn neurons and primary sensory neurons at normal state. In contrast to our previous results that upregulation of CK1ϵ protein expression was observed in the spinal dorsal horn (L5) and injured L5 DRG neurons ipsilateral to the nerve injury in the mouse L5/6 spinal nerve injury model [12], we could not detect significant increases of protein expression levels of CK1α, δ, ϵ isoforms in the spinal cord (L4-6) and DRGs (L4-6) in the present immunoblot study as shown in Additional file 1. It would be, therefore, interesting to hypothesize that activity of CK1 in the primary sensory neurons and/or spinal dorsal horn neurons regulated by the peripheral inflammation would contribute to the spinal plasticity which has an important role in generating inflammatory pain states. Several mechanisms, such as control of subcellular localization by regulating membrane and/or nuclear trafficking, and modulation of the inhibitory autophosphorylation sites located at C-terminal domains of CK1, which have been identified to modulate CK1 activity in other experimental conditions [5,6], might also be relevant to our present observation. Targeting mechanisms that counter-regulate the spinal consequences of peripheral inflammation by CK1 inhibitors or other methods may provide an effective way to control chronic pain. Further elucidation of CK1 signaling mechanisms including spatial distribution of CK1 isoforms before and after inflammation is considered to be critical in future clinical development for directing the signaling pathways with small molecule agents.

## Conclusions

In summary, the present study suggests an important role of CK1 in inflammatory pain symptoms. Although the specific role of each CK1 isoforms in inflammatory pain remains elusive, CK1 inhibitors could be promising new therapeutics for treating pain associated with inflammation as well as neuropathic pain.

## Methods

### In vitro kinase assay

The inhibitory effects of TG003 and IC261 against CK1 isoforms were tested using the QuickScout screening assist mobility shift assay with the ATP concentration at the *Km* (4.1 μM for CK1α, 6.3 μM for CK1γ1, 10 μM for CK1γ2, 3.2 μM for CK1γ3, 7.7 μM for CK1δ, and 16 μM for CK1ϵ; Carna Biosciences, Inc., Kobe, Japan). Detailed information on the assay condition is available on the website of Carna Biosciences (http://www.carnabio.com). Full-length human CK1α, CK1γ1, CK1γ2, CK1γ3 and catalytic domain of human CK1ϵ were expressed as N-terminal GST-fusion protein using baculovirus system, and purified by using glutathione sepharose chromatography. Catalytic domain of CK1δ was expressed as N-terminal GST-fusion protein in *E. coli*, and purified by using glutathione sepharose chromatography.

### Vector construction

PCR-amplified fragments of mCherry (Clontech) and PER3 (Accession: NP_058515) were fused in-frame by overlap-extension PCR method to generate mCherry-PER3, respectively, as described previously [43] with some modifications. The combined fragment was inserted into pCAGIPuro vector, an IRES-based bicistronic expression vector where the gene of interest and a puromycin resistant gene are expressed from a single mRNA, which enables almost all of the cells selected with puromycin to express the gene product. PCR-amplified fragments of FLAG-tagged CK 1δ (Accession: BC015775) and ϵ (Accession: BC006490) were fused in-frame to the amino-terminus of EGFP via F2A peptide sequence by overlap-extension PCR method, which enables bicistronic expression of FLAG-tagged CK1 isoforms and EGFP. The combined fragments were inserted into pcDNA5/FRT/TO (Life Technologies). The reconstituted vector sequences are available upon request.

### Cell culture and transfection

Flp-In/T-REx HEK293 cell (Life Technologies) was maintained in low glucose Dulbecco's modified Eagle's medium (Nacalai Tesque, Kyoto, Japan) supplemented with 10% fetal bovine serum (Nichirei Biosciences, Tokyo, Japan), 100 units/ml of penicillin and 100 μg/ml of streptomycin (Nacalai Tesque). Cells were transfected with plasmid DNAs using polyethylenimine MAX (Polysciences) as described previously [44], and then selected with hygromycin B (Life Technologies) for pcDNA5/FRT/TO vectors and puromycin (Nacalai Tesque) for pCAGIPuro vectors to establish the stable cell lines.

### PER3 nuclear translocation assay

HEK293 cells seeded in a density of 1 × 10^5^ cells/dish in polyethyleneimine-coated 35 mm glass bottom dishes (MatTek, Ashland, MA) were cultured for 2 days. Cells were pre-incubated with 0.1% dimethyl sulfoxide (DMSO) containing 30 μM TG003, 30 μM TG001, or 1 μM PF-670462 (Merck, Darmstadt, Germany) for 1 hour at 37°C before expression of CK1δ or CK1ϵ was induced with 1 μg/ml of doxycycline. After 8 hour incubation with doxycycline at 37°C, cells were fixed with 10% Formaldehyde Neutral Buffer Solution (Nacalai Tesque) for 10 min at room temperature. Cells were washed twice with PBS and then stained with 5 μg/ml of Hoechst33342 (Dojindo, Kumamoto, Japan) in PBS for 30 min at room temperature. The Hoechst33342 solution was removed and cells were washed with PBS, and stored in 1.5 ml PBS at 4°C in the dark until taking fluorescent images on the Confocal Laser Scanning Biological Microscope FV10i (Olympus, Tokyo, Japan).

The fluorescent images were analyzed by the compartmental analysis algorithm predefined in Cellomics BioApplications (Thermo Fisher Scientific, Waltham, MA). The nuclear-cytoplasmic ratio of the mCherry-PER3 signal intensity was quantified by dividing the mean average mCherry intensity in the nuclear area defined as “circ” by the mean average mCherry intensity of a “ring” around this area, which covered a cytoplasmic region. The distance of the circ to the nuclear outline was 16 pixels. The ring had a width of 4 pixels and a distance of 1 pixel from the nuclear outline. The fluorescent image containing over 15 objects (cells) counted by the compartmental analysis algorithm was used for analysis. The objects that were under 650 of the mean average EGFP intensity in the nuclear area were excluded. Analysis data was exported into Excel file for statistical analysis.

### Animals

Male C57BL/6 J mice (5 weeks old) were purchased from Clea Japan, Inc. (Tokyo, Japan) and housed under controlled temperature (24 ± 1°C) and humidity (55 ± 10%) with a 12-hour light-dark cycle with food and water freely available. The animal experiments were approved by the Animal Care Committees of Tokyo Medical and Dental University (approval No. 0090173) and Kagoshima University (approval No. MD10053), and were conducted in accordance with the ethical guidelines for the study of experimental pain in conscious animals published by the International Association of the Study of Pain (1995) [45] and the European Communities Council Directive of 24 November, 1986 (86/609/EEC).

### Animal models and behavioral studies

To produce acute and persistent inflammatory pain, carrageenan (2% lambda carrageenan in saline, 25 μl, Sigma, St. Louis, MO) and complete Freund’s adjuvant (CFA, 25 μl, Sigma) were injected into the plantar surface of the right hindpaw under light halothane anesthesia, respectively [46-49]. Control mice were treated with saline or incomplete Freund’s adjuvant (IFA, Sigma), respectively. Mechanical allodynia and thermal hyperalgesia were measured using the Dynamic Plantar Aesthesiometer (Ugo Basile, Comerio VA, Italy) and the Paw Thermal Stimulator (UCSD, San Diego, CA, USA), respectively as described [12]. In CFA model, these behavioral experiments were conducted 3 days after the injection. Intrathecal (i.t.) injection was given in a volume of 5 μl by percutaneous puncture through an intervertebral space at the level of the 5th or 6th lumbar vertebra, according to a previously reported procedure [12,50]. An investigator, who was unaware of the drug treatment, performed all of the behavioral experiments.

### Immunoblot analysis

Six hours after carrageenan or saline injection, and 3 days after CFA or IFA injection, mice were anesthetized with sodium pentobarbital (50 mg/kg), and the lumbar spinal cord and DRGs (L4-L6) were quickly removed. Each sample was homogenized in a lysis buffer [150 mM NaCl, 1 mM EDTA, 1% NP-40, 0.5% sodium deoxycholate, 0.1% SDS, 1:100 diluted protease inhibitor cocktail (Sigma), and 50 mM Tris-HCl, pH 8.0]. Protein concentrations were determined with a Bio-Rad protein assay kit (Bio-Rad, Hercules, CA). Proteins were separated by SDS-PAGE (7.5% gel) and then transferred to a polyvinylidene difluoride membrane (Millipore, Billerica, MA). Anti-CK1α (rabbit polyclonal, raised against amino acids 281-337 at the C-terminus of human CK1α; 1: 2,000; no. sc-28886, Santa Cruz Biotechnology, Santa Cruz, CA), anti-CK1ϵ (rabbit polyclonal, raised against amino acids 301-360 near the C-terminus of human CK1ϵ; 1: 1,000; no. sc-25423, Santa Cruz Biotechnology) and anti-CK1δ antibody (rabbit polyclonal, NC10, 1:4,000; kindly donated by Prof. Uwe Knippschild, Univ. Ulm, Germany) were used. The specificities of the three antibodies were characterized and reported previously in several studies including ours [12,41,42,51]. We have also conducted control staining experiments; omission of primary antibody or secondary antibody, and substitution of primary antibody with normal rabbit IgG. We did not obtain any signals from these control experiments (data not shown).

Immunoreactivity was detected by using the ECL system (GE Healthcare, Buckinghamshire, UK). An anti-glyceraldehyde-3-phosphate dehydrogenase (GAPDH) antibody (mouse monoclonal, 1:20,000; no. MAB374, Chemicon, Temecula, CA) or β-actin (mouse monoclonal, 1:1,000; no. sc-47778, Santa Cruz Biotechnology) were used to normalize protein loading. Relative intensities of the bands were quantified by using an image analysis system with Image J software, version 1.40 g (National Institutes of Health, Bethesda, MD). At least two independent immunoblot experiments of three independent spinal cord and DRG samples were analyzed.

### Patch-clamp recordings from spinal dorsal horn neurons

Adult mouse spinal cord slices were prepared according to the method of Yoshimura & Jessell [21,22]. Briefly, 6 hours after carrageenan or saline injection, and 3 days after CFA or IFA injection, transverse slices (thickness, 800-900 μm) of the L5 spinal segments with the L5 dorsal root attached were cut on a vibrating blade microtome. The slices were superfused with Krebs solution (10-15 ml/min) saturated with 95% O_2_ and 5% CO_2_ at 36 ± 1°C. The composition of Krebs solution was as follows (in mM): NaCl 117; KCl 3.6; NaHCO_3_ 25; NaH_2_PO_4_ 1.2; CaCl_2_ 2.5; MgCl_2_ 1.2, and glucose 11 (pH 7.4 after gas saturation).

Blind whole-cell patch-clamp recordings were made from the lamina II (substantia gelatinosa: SG) neurons ipsilateral to carrageenan, CFA, or vehicle (saline or IFA) injection in voltage clamp mode. Patch pipettes were fabricated from thin-walled, borosilicate, glass-capillary tubing (1.5 mm o.d., World Precision Instruments). After establishing the whole-cell configuration, neurons were held at the potential of -70 mV to record spontaneous excitatory postsynaptic currents (sEPSCs) and at the potential of 0 mV to record spontaneous inhibitory postsynaptic currents (sIPSC). Under these conditions, GABA- and glycine-mediated IPSCs and glutamate-mediated EPSCs, respectively, were negligible, because these holding potential were close to the reversal potentials of IPSCs and EPSCs, respectively [52]. Recording electrodes were filled with either potassium gluconate-based solution (in mM: K-gluconate 135; KCl 5; CaCl_2_ 0.5; MgCl_2_ 2; EGTA 5; HEPES 5; ATP-Mg 5; adjusted with KOH to pH 7.2) to investigate EPSCs, or Cs-based solution (in mM: Cs_2_SO_4_ 110; tetraethylammonium 5; CaCl_2_ 0.5; MgCl_2_ 2; EGTA 5; HEPES 5; ATP-Mg 5; adjusted with CsOH to pH 7.2) to examine IPSCs. The resistance of a typical patch pipette is 5-10 MΩ. Membrane currents were amplified with an Axopatch 200B amplifier (Molecular Devices, Sunnyvale, CA, USA) in voltage-clamp mode. Signals were low-pass filtered at 5 kHz and digitized at 333 kHz with an A/D converter (Digidata 1322, Molecular Devices). Data were stored with a personal computer using pCLAMP10 software and analyzed with Mini Analysis software (Synaptosoft Inc., Decatur, GA, USA).

The average values of both frequency and amplitude of sEPSCs or sIPSCs during the control (1 min) and 5-10 min after the drug application (1 min period after the attainment of steady effect of each drug) were calculated and quantified as relative changes in frequency and amplitude. Since the characteristics of sEPSCs and sIPSCs parameters (frequency and amplitude) were not significantly different among naïve-, saline- and IFA-control, data from each control were combined.

### Drugs

IC261 was from Calbiochem, LaJolla, CA, USA. PF-670462 was obtained from Tocris bioscience, Bristol, UK. TG003 and TG001 were synthesized according the procedures described previously [13]. These drugs were made up as concentrated stock solution in DMSO, aliquoted and stored at –20°C. An aliquot was diluted to the desired concentration in saline or Krebs solution immediately prior to use. The dose ranges of IC261 and TG003 used were determined according to our previous report (for IC261) [12] and preliminary study (for TG003).

### Statistical analysis

Experimental data are expressed as mean ± SEM. Single comparisons were made using Student’s two-tailed paired or unpaired *t*-test. One-way ANOVA followed by the Dunnett’s or Tukey’s test was used for multiple comparisons. *P* < 0.05 was considered statistically significant.

## Abbreviations

CFA: Complete Freund’s adjuvant; CK: Casein kinase; Clk: cdc2-like kinase; DRG: Dorsal root ganglion; GAPDH: Glyceraldehyde-3-phosphate dehydrogenase; IFA: Incomplete Freund’s adjuvant; i.t.: Intrathecal; sEPSC: Excitatory and inhibitory postsynaptic currents; sIPSC: Inhibitory postsynaptic currents; SG: Substantia gelatinosa.

## Competing interests

The authors declare that they have no competing interests.

## Authors’ contributions

TK participated in the design of the study, performed behavioral and electrophysiological studies and wrote the manuscript. ES, TA and DK carried out behavioral and immunoblot analysis. MT and IK performed in vitro kinase assay and molecular biological study and wrote the manuscript. TT, MY, MH and AM participated in the design of the study and reviewed the manuscript. All authors read and approved the final manuscript.

## Supplementary Material

Additional file 1 **Carrageenan- and CFA-induced inflammation did not upregulate CK1α, δ and ϵ expression.** Immunoblot analyses of CK1α **(A)**, δ **(B)** and ϵ **(C)** expression levels in the spinal cord and DRGs. L4-6 spinal segments and DRGs ipsilateral to the inflammation were dissected 6 hours after carrageenan (Car) and 3 days after CFA injection. As a control, saline (Sal) and incomplete Freund’s adjuvant (IFA) were injected instead of Car and CFA, respectively. Click here for file

Additional file 2 Comparison of passive membrane properties among L5 SG neurons obtained from control and inflamed mice. Click here for file

Additional file 3 **Effects of inflammation on spontaneous EPSCs (sEPSCs, A) and IPSCs (sIPSCs, B).** Hindpaw injection of CFA but not carrageenan (Car) increased mean frequency of sEPSCs and decreased mean frequency of sIPSCs. Neither CFA nor carrageenan changed mean amplitudes of sEPSCs and sIPSCs. Three days (CFA 3d) or 6 hours (Car 6 h) after injection, spinal cord slices were prepared and blind whole-cell patch-clamp recordings were made from the SG neurons ipsilateral to Car, CFA, or vehicle injection. *P < 0.05, **P < 0.01; one-way ANOVA followed by Tukey’s post hoc test. Click here for file
