# Supplementary material for: Alleviation of behavioral hypersensitivity in mouse models of inflammatory pain with two structurally different casein kinase 1 (CK1) inhibitors
Source: Mol Pain. 2014 Mar 10;10:17. doi: 10.1186/1744-8069-10-17 (PMC4008364; doi:10.1186/1744-8069-10-17)
Supplement: Additional file 1 — Carrageenan- and CFA-induced inflammation did not upregulate CK1α, δ and ϵ expression. Immunoblot analyses of CK1α (A), δ (B) and ϵ (C) expression levels in the spinal cord and DRGs. L4-6 spinal segments and DRGs ipsilateral to the inflammation were dissected 6 hours after carrageenan (Car) and 3 days after CFA injection. As a control, saline (Sal) and incomplete Freund’s adjuvant (IFA) were injected instead of Car and CFA, respectively. [file 1744-8069-10-17-S1.pdf]

Additional file 1: Carrageenan- and CFA-induced inflammation did not upregulate CK1α, δ and ε expression. Immunoblot analyses of CK1α **(A)**, δ **(B)** and ε **(C)** expression levels in the spinal cord and DRGs. L4-6 spinal segments and DRGs ipsilateral to the inflammation were dissected 6 hours after carrageenan (Car) and 3 days after CFA injection. As a control, saline (Sal) and incomplete Freund's adjuvant (IFA) were injected instead of Car and CFA, respectively.

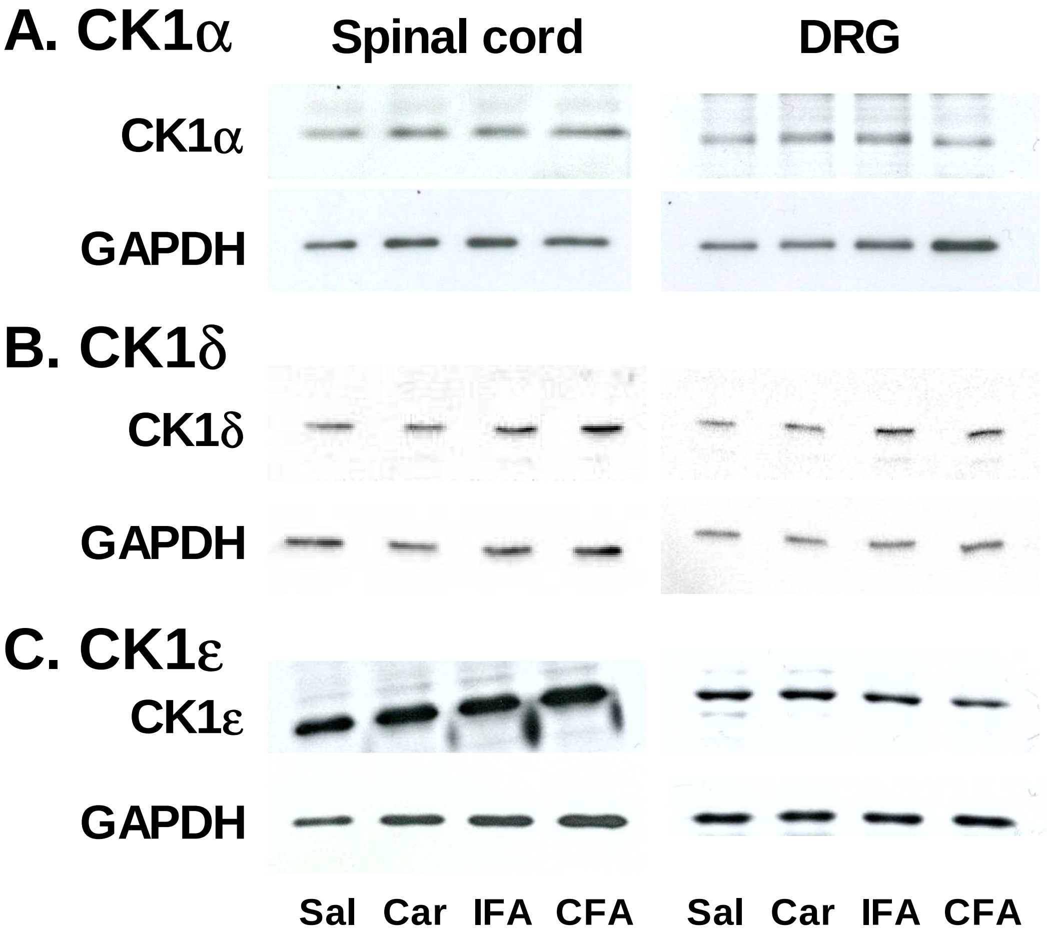

Kurihara et al. Additional file 1
