# Supplementary material for: Alleviation of behavioral hypersensitivity in mouse models of inflammatory pain with two structurally different casein kinase 1 (CK1) inhibitors
Source: Mol Pain. 2014 Mar 10;10:17. doi: 10.1186/1744-8069-10-17 (PMC4008364; doi:10.1186/1744-8069-10-17)
Supplement: Additional file 2 — Comparison of passive membrane properties among L5 SG neurons obtained from control and inflamed mice. [file 1744-8069-10-17-S2.pdf]

Additional file 2. Comparison of passive membrane properties among L5 SG neurons obtained from control and inflamed mice.

	RMP (mV)	IMR (MΩ)
Naïve	- 66.4 ± 1.44 (*n* = 17)	636.7 ± 114.2 (*n* = 7)
Saline 6 h	- 66.7 ± 1.35 (*n* = 17)	664.7 ± 55.3 (*n* = 16)
Carrageenan 6 h	- 63.9 ± 1.04 (*n* = 32)	713.2 ± 49.4 (*n* = 12)
IFA 3d	- 67.8 ± 2.23 (*n* = 9)	614.1 ± 76.4 (*n* = 7)
CFA 3d	- 62.9 ± 1.05 (*n* = 44)	566.2 ± 52.5 (*n* = 26)

Carrageenan and CFA inflammation did not change resting membrane potential (RMP) or input membrane resistance (IMR). Spinal cord slices were prepared 6 hours after carrageenan or 3 days after CFA injection and blind whole-cell patch-clamp recordings were made from the SG neurons ipsilateral to carrageenan or CFA injection. As a control, saline and incomplete Freund's adjuvant (IFA) were injected instead of carrageenan and CFA, respectively. Each value represents the mean ± SEM.

Kurihara et al. Additional file 2
