# Supplementary material for: Alleviation of behavioral hypersensitivity in mouse models of inflammatory pain with two structurally different casein kinase 1 (CK1) inhibitors
Source: Mol Pain. 2014 Mar 10;10:17. doi: 10.1186/1744-8069-10-17 (PMC4008364; doi:10.1186/1744-8069-10-17)
Supplement: Additional file 3 — Effects of inflammation on spontaneous EPSCs (sEPSCs, A) and IPSCs (sIPSCs, B). Hindpaw injection of CFA but not carrageenan (Car) increased mean frequency of sEPSCs and decreased mean frequency of sIPSCs. Neither CFA nor carrageenan changed mean amplitudes of sEPSCs and sIPSCs. Three days (CFA 3d) or 6 hours (Car 6 h) after injection, spinal cord slices were prepared and blind whole-cell patch-clamp recordings were made from the SG neurons ipsilateral to Car, CFA, or vehicle injection. *P < 0.05, **P < 0.01; one-way ANOVA followed by Tukey’s post hoc test. [file 1744-8069-10-17-S3.pdf]

Additional file 3. Effects of inflammation on spontaneous EPSCs (sEPSCs, A) and IPSCs (sIPSCs, B). Hindpaw injection of CFA but not carrageenan (Car) increased mean frequency of sEPSCs and decreased mean frequency of sIPSCs. Neither CFA nor carrageenan changed mean amplitudes of sEPSCs and sIPSCs. Three days (CFA 3d) or 6 hours (Car 6h) after injection, spinal cord slices were prepared and blind whole-cell patch-clamp recordings were made from the SG neurons ipsilateral to Car, CFA, or vehicle injection. $^{*}P < 0.05$, $^{**}P < 0.01$; one-way ANOVA followed by Tukey's post hoc test.

A

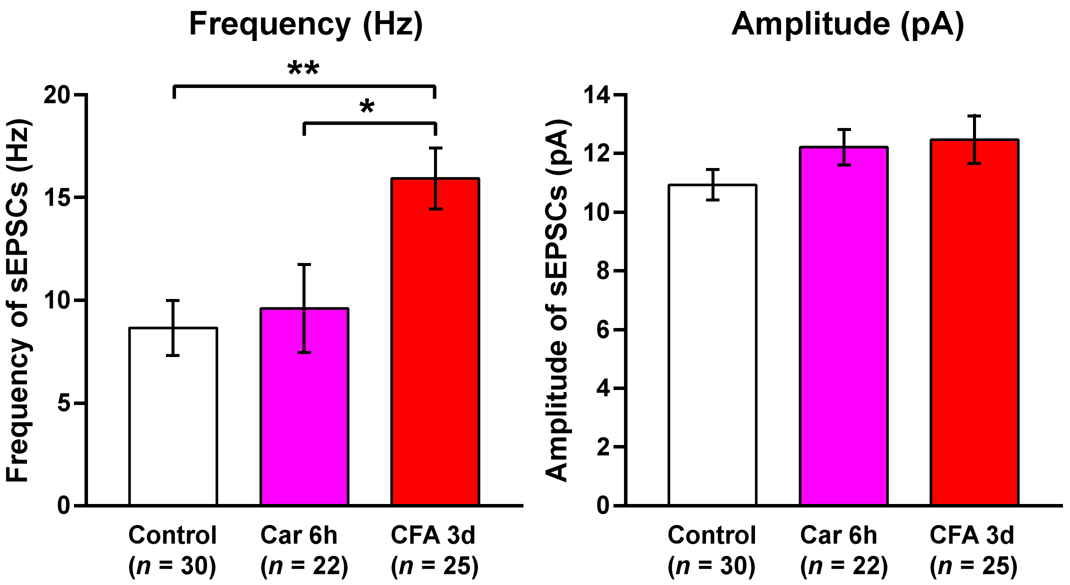

B

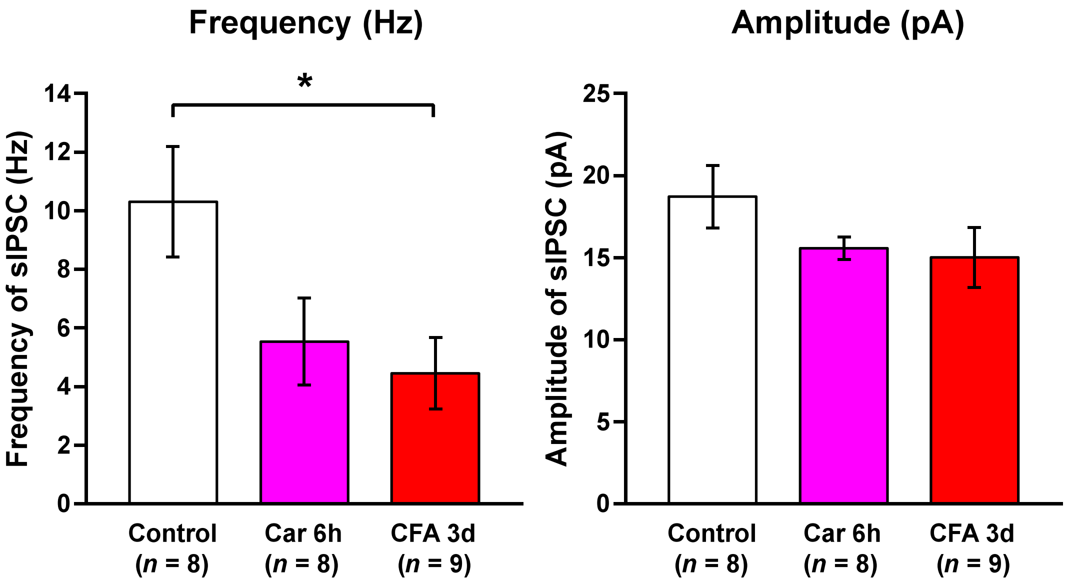

Kurihara et al. Additional file 3
